# Using Potassium Bicarbonate to Improve the Water-Holding Capacity, Gel and Rheology Characteristics of Reduced-Phosphate Silver Carp Batters

**DOI:** 10.3390/molecules28145608

**Published:** 2023-07-24

**Authors:** Chun Xie, Bei-Bei Shi, Guang-Hui Liu, Si-Han Li, Zhuang-Li Kang

**Affiliations:** 1 School of Pharmacy, Shangqiu Medical College, Shangqiu 476100, China; 18115152505@163.com (B.-B.S.); lgh1178@163.com (G.-H.L.); sqyzjwczyrd2022@163.com (S.-H.L.); 2 Engineering Research Center for Huaiyang Cuisin of Jiangsu Province, College of Tourism and Culinary, Yangzhou University, Yangzhou 225127, China; kzlnj1988@163.com

**Keywords:** reduced phosphate, potassium bicarbonate, cooking yield, secondary structure, textural

## Abstract

To study the use of partial or total potassium bicarbonate (PBC) to replace sodium tripolyphosphate (STPP) on reduced-phosphate silver carp batters, all the batters were composed of silver carp surimi, pork back fat, ice water, spices, sugar, and sodium chloride. Therein, the sample of T1 contained 4 g/kg STPP; T2 contained 1 g/kg PBC, 3 g/kg STPP; T3 contained 2 g/kg PBC, 2 g/kg STPP; T4 contained 3 g/kg PBC, 1 g/kg STPP; T5 contained 4 g/kg PBC, and they were all produced using a bowl chopper. The changes in pH, whiteness, water- and oil-holding capacity, gel and rheological properties, as well as protein conformation were investigated. The pH, cooking yield, water- and oil-holding capacity, texture properties, and the G′ values at 90 °C of the reduced-phosphate silver carp batters with PBC significantly increased (*p* < 0.05) compared to the sample without PBC. Due to the increasing pH and enhanced ion strength, more β-sheet and β-turns structures were formed. Furthermore, by increasing PBC, the pH significantly increased (*p* < 0.05) and the cooked silver carp batters became darkened. Meanwhile, more CO_2_ was generated, which destroyed the gel structure, leading the water- and oil-holding capacity, texture properties, and G′ values at 90 °C to be increased and then decreased. Overall, using PBC partial as a substitute of STPP enables reduced-phosphate silver carp batter to have better gel characteristics and water-holding capacity by increasing its pH and changing its rheology characteristic and protein conformation.

## 1. Introduction

There are more and more exogenous phosphorus additives in food at present, for example, bread, coke, meat, and fish products. It is easy to consume excessive amounts of phosphorus and destroy the balance of calcium and phosphorus in the body, leading to bone, kidney, and other diseases [1,2]; thus, reduced-phosphate foods are becoming more and more popular with consumers. Surimi products, such as fish balls, fish steak, and crab meat sticks, are a kind of high-quality, nutritious, and convenient high-protein food, as they have a good cost performance and are widely accepted by consumers [3,4]. To improve the processing characteristics of surimi products, food additives containing phosphorus (sodium tripolyphosphate—STPP, sodium pyrophosphate—SPP, and sodium hexametaphosphate—SHMP) are often added [5]. The functions of food additives containing phosphorus in the processing of surimi products are as follows: increasing pH, ionic strength, and chelate metal ions; dissociating actomyosin and inducing more protein structure changes [6,7]. A direct reduction in phosphorous reduces the water-holding capacity, texture properties, and shelf life of surimi products [8,9]. Meanwhile, food additives containing phosphorus are cheap, effective, and easily handled. For these reasons, it is difficult to find alternatives to food additives containing phosphorus.

Potassium bicarbonate (PBC) is a kind of acidity corrector which is approved by the Food and Drug Administration (FDA) with the Generally Recognized as Safe status, is appropriate for use in foods (FDA, 2016), and decomposes into HCO_3_^−^, K^+^, OH^−^, etc., in an aqueous solution. It is cheaper and more easily soluble in water than food additives containing phosphorus, and used in meat and meat products, dough, and other foods to improve their processing characteristics and flavor [10,11,12,13]. Mohan, Jaico, Kerr, and Singh [13] reported that PBC and sodium bicarbonate with or without sodium chloride improved the water-holding capacity of processed ground beef, and the sample with PBC and sodium chloride had the best adhesiveness values. Lee, Sharma, Brown, and Mohan [12] found that the breast meat marinated with PBC and sodium bicarbonate had greater water retention and cooked product qualities, and that PBC is a healthier alternative to phosphate or sodium bicarbonate when marinating chicken breast meat. Varo, Martin-Gomez, Serratosa, and Merida [10] concluded that PBC can change organoleptic characteristics, reduce the acidity of blueberry wine, and improve its quality. As far as we know, there remain some unknowns regarding the water-holding capacity, gel characteristics, and protein conformation of reduced-phosphate silver carp surimi with the use of PBC to partially and totally replace food additives containing phosphorus. Therefore, this study aimed to study the effect of PBC and STPP (the ratio of PBC and STPP of T1, T2, T3, T4, and T5 were 4:0, 3:1, 2:2, 1:3, and 0:4, respectively) on the pH, gel and rheological characteristics, and protein conformation of silver carp surimi, in order to find a new method to improve the quality of reduced-phosphate silver carp surimi products.

## 2. Results and Discussion

### 2.1. pH

**Figure 1:**
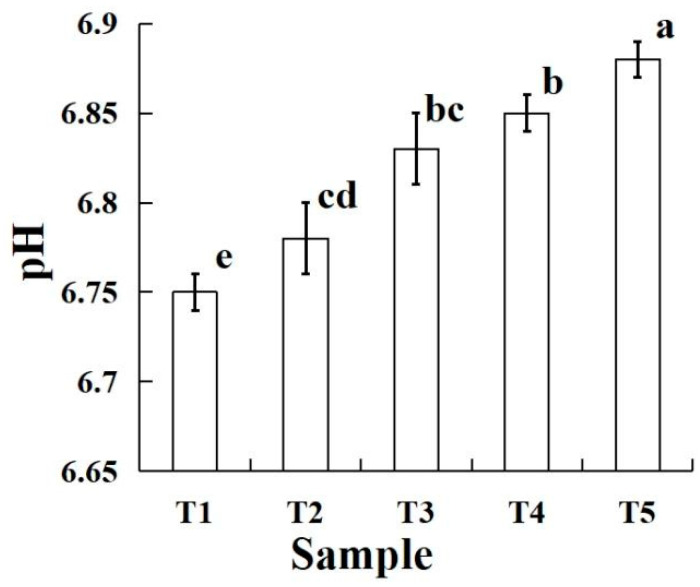
pH of the raw silver carp batters made with various amounts of potassium bicarbonate and sodium tripolyphosphate. T1 contained sodium tripolyphosphate 0.4 g; T2 contained potassium bicarbonate 0.1 g and sodium tripolyphosphate 0.3 g; T3 contained potassium bicarbonate 0.2 g and sodium tripolyphosphate 0.2 g; T4 contained potassium bicarbonate 0.3 g and sodium tripolyphosphate 0.1 g; T5 contained potassium bicarbonate 0.4 g. Each value represents the mean ± SD, *n* = 4. ^a–e^ Different parameter superscripts in the table indicate significant differences (*p* < 0.05).

The effects of PBC and STPP on the pH of raw silver carp batters are shown in Figure 1. The pH of raw silver carp batter with PBC significantly increased (*p* < 0.05) from approximately 6.75 to 6.88 compared to the sample of T1. PBC and STPP are strong alkalies and weak acid salts, but PBC has a better buffer capacity than STPP. Mohan, Jaico, Kerr, and Singh [13] found that adding PBC and sodium bicarbonate alone with or without sodium chloride could shift the pH of ground beef without a significant difference. Varo, Martin-Gomez, Serratosa, and Merida [10] reported that the pH of blueberry wine with PBC (1 g/L) increased by approximately 0.4–0.6 units. A similar result found that the pH of raw chicken batters with other bicarbonates (0.3% and 0.5% sodium bicarbonate) causes an increase of approximately 0.13 units compared to the samples with 0.3% and 0.5% STPP [9]. Petracci et al. [14] used bicarbonate and STPP to marinade the broiler breast meat, and they found that the pH of the samples was increased by approximately 0.70 and 0.30 units, respectively. In addition, the pH of raw silver carp batters significantly increased (*p* < 0.05) with increasing PBC, except for the samples of T2 and T3, T3, and T4. A possible reason for this is that the raw silver carp batter has a good buffering capacity; when the replacement amount of PBC was not enough, the pH difference between the adjacent samples was not significant [15]. Thus, the use of PBC to replace STPP could increase the pH of raw silver carp batter.

### 2.2. Emulsion Stability

Emulsification stability reflects the water- and oil-retention ability of raw silver carp batter. The higher the stability, the stronger the water and oil retention. The changes in PBC and STPP on the emulsion stability of raw silver carp batters are shown in Table 1. Compared to the sample of T1, the total fluid, water, and fat released by the raw silver carp batter with PBC significantly decreased (*p* < 0.05) from approximately 12.80% to 8.36%, from 9.92% to 6.84%, and from 2.63% to 1.39%, respectively. On the other, the total fluid, water, and fat released by the raw silver carp batter significantly decreased (*p* < 0.05) with increasing PBC, except for the T2 and T5, T3 and T4 samples. Increasing PBC can shift the pH and increase the ionic strength and buffering capacity of raw silver carp batter, leading to more salt-soluble proteins swelled and dissolved [9] and wrapped around fat particles, forming good protein network structures during the heating process, thereby improving the ability of raw silver carp batter to bind water and oil [16,17]. Especially, due to PBC’s ability to produce large amounts of carbon dioxide during the process of heating, the gas destroys the gel structure, causing the water- and oil-retention ability of silver carp batter to decrease [18,19]. Therefore, the use of PBC to replace STPP could improve the emulsion stability of raw silver carp batter.

### 2.3. Cooking Yield

**Figure 2:**
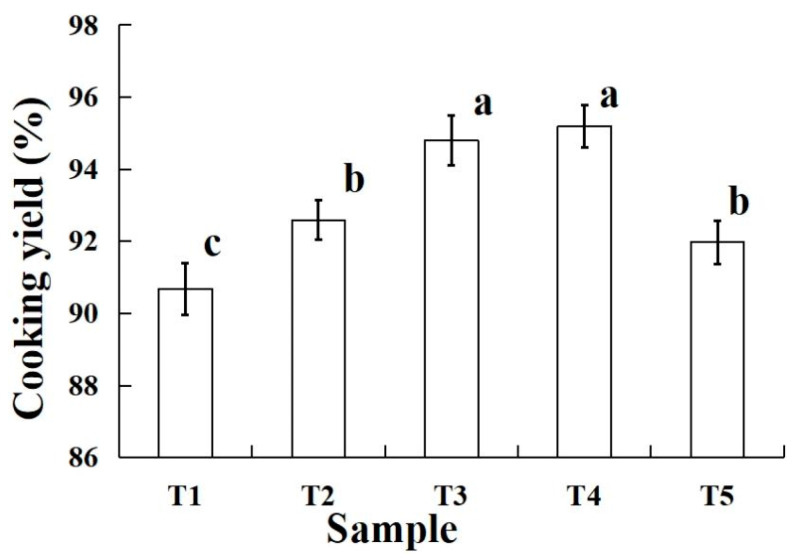
Cooking yield of the raw silver carp batters made with various amounts of potassium bicarbonate and sodium tripolyphosphate. T1 contained sodium tripolyphosphate 0.4 g; T2 contained potassium bicarbonate 0.1 g and sodium tripolyphosphate 0.3 g; T3 contained potassium bicarbonate 0.2 g and sodium tripolyphosphate 0.2 g; T4 contained potassium bicarbonate 0.3 g and sodium tripolyphosphate 0.1 g; T5 contained potassium bicarbonate 0.4 g. Each value represents the mean ± SD, *n* = 4. ^a–c^ Different parameter superscripts in the table indicate significant differences (*p* < 0.05).

The changes in the cooking yield of raw silver carp batter in various amounts of PBC and STPP are shown in Figure 2. The cooking yield of the raw silver carp batter with PBC significantly increased (*p* < 0.05) compared to the T1 sample. It is possible that more salt-solution protein was extracted after adding PBC because it shifted the pH and enhanced the ionic strength [20,21]. In particular, when shifting the isoelectric point (PI) of myofibrillar protein, the ability to bind and retain water was increased, thus improving the ability of the silver carp batter to prevent water from spreading outside [22,23]. Meanwhile, the cooking yield of raw silver carp batter significantly increased (*p* < 0.05) with increasing PBC, except for the T2 and T5, T3 and T4 samples. The main reason for this is that the gel structure was destroyed by carbon dioxide gas during heating when excess PBC was added [17,21]. Overall, the excessive addition of PBC is not conducive to improvement in cooking rate.

### 2.4. Texture Properties

Texture profile analysis is widely used to estimate the texture characteristics of gel products. The changes in texture profile analysis of cooked silver carp batters with various amounts of PBC and STPP are shown in Table 2. The hardness, springiness, adhesiveness, and chewiness of cooked silver carp batter with PBC significantly increased (*p* < 0.05) compared to the T1 sample. Due to the enhanced pH and ionic strength of silver carp batter after adding PBC, the interactions of protein, water, and fat were increased [9,15,22]. Kang et al. [23] showed that the larger-sized protein aggregations of myofibrillar protein promote the formation of a stable and elastic gel matrix. Meanwhile, the hardness, springiness, adhesiveness, and chewiness of cooked silver carp batter significantly increased (*p* < 0.05) with increasing PBC, except for the T2 and T5, T3 and T4 samples. Some papers have found that added bicarbonate (sodium bicarbonate or PBC) enhances the texture properties of emulsion products; however, the gel structure was destroyed by carbon dioxide, which produced from sodium bicarbonate or PBC, leading to the texture properties being decreased [13,23].

### 2.5. Whiteness

Whiteness, as an important factor in fish food quality, is the first sensory feature of consumer evaluation. The changes in the whiteness of the cooked silver carp batters were made with various amounts of PBC and STPP, as shown in Table 2. Compared to the T1 sample, the whiteness of cooked silver carp batter with PBC significantly decreased (*p* < 0.05) from approximately 88.71 to 74.26, and it significantly decreased (*p* < 0.05) with the increase in PBC. Due to the fact that increasing pH lowers the oxidation rate of myoglobin to metmyoglobin [24], some researchers have found that the color of sea and meat products becomes dark-brown with the increase in bicarbonate content [18,25,26]. A similar study was reported by Lee, Sharma, Brown, and Mohan [12], who found that the marination of chicken breast meat with 1.5% PBC resulted in more darkening as compared to the sample with 0.5% PBC, along with the a* value being decreased and the b* value being increased. Åsli and Mørkøre [26] found that the L* value of salted Atlantic cod was decreased upon bicarbonate addition. Wu et al. [27] reported that the concentration of bicarbonate affected the color of pork myofibrillar protein, and the whiteness decreased by increasing the bicarbonate content. This result indicated that adding PBC can reduce the denaturation of myoglobin and darken the color of cooked silver carp batters.

### 2.6. Rheological Properties

**Figure 3:**
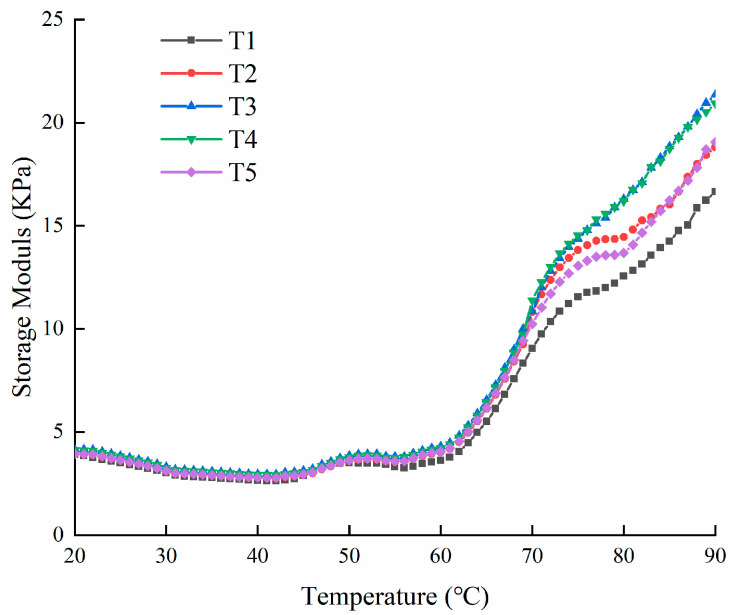
Changes in storage modulus (G′) of the raw silver carp batters from 20 °C to 90 °C made with various amounts of potassium bicarbonate and sodium tripolyphosphate. T1 contained sodium tripolyphosphate 0.4 g; T2 contained potassium bicarbonate 0.1 g and sodium tripolyphosphate 0.3 g; T3 contained potassium bicarbonate 0.2 g and sodium tripolyphosphate 0.2 g; T4 contained potassium bicarbonate 0.3 g and sodium tripolyphosphate 0.1 g; T5 contained potassium bicarbonate 0.4 g.

The G′ can reflect the gradual change in myosin and actin of fish or meat [28]. The changes in G′ from 20 to 90 °C in raw silver carp batters with various amounts of PBC and STPP are shown in Figure 3. They all have rheological curves with three stages during the heating process. In the first stage, a slow downward trend was observed from 20 °C to 42 °C, which may be due to the pork back fat melting; moreover, silver carp surimi contains a high level of proteases, which can induce the degradation of myofibrillar proteins associated with gel weakening [29]. Following this, a slight upward trend was noticed from 43 °C to 50 °C (T1), and from 43 °C to 52 °C (T2, T3, T4, and T5), which was caused by the protein–protein interactions and formed a weak gel [30]. The reason for this difference is that the silver carp batters with PBC promote more salt-soluble proteins to dissolve compared to the sample with STPP, in which the high protein solubility facilitates the cross-linking between proteins [19]. In the second stage, the myosin tails were denatured and this destroyed the gel network formed in the first stage, leading to a moderate decrease in G′ found from 51 °C to 56 °C (T1) and from 53 °C to 55 °C (T2, T3, T4, and T5). This result indicates that, compared to STPP, PBC can enhance the thermal stability of the myosin tail and reduce the effect of denaturation on the gel structure [31,32]. In the third stage, due to the transformation of silver carp batter from a sticky sol state to an elastic gel structure, the continuous increase in G′ was observed from 57 °C to 90 °C (T1) and from 56 °C to 90 °C (T2, T3, T4, and T5) [33,34]. Moreover, all the G′ values of the samples at 90 °C with PBC were higher compared to the sample with STPP, and T3 and T4 had the highest G′ values at 90 °C (16.3 kPa), implying that the textural properties of silver carp batters with PBC significantly increased.

### 2.7. Raman Spectroscopic

The secondary structure of the protein is sensitive to pH, ion strength, and temperature, and the α-helice, β-sheet, β-turns, and random coil structures can be transformed with each other [35,36]. The changes in α-helice, β-sheet, β-turns, and random coil structures of cooked silver carp batters with various amounts of PBC and STPP are shown in Table 3. The α-helice and random coil structures content significantly decreased (*p* < 0.05), along with the β-sheet and β-turns structures content which significantly increased (*p* < 0.05) when PBC was added. PBC and STPP are alkaline, which has an influence on the protein structure and breaks the bonds between proteins [37]. Due to the pH and ion strength of cooked silver carp batters with PBC being higher than the sample with STPP, when using PBC to replace STPP, the α-helix structure unfolds, gradually transforming into β-sheet and β-turns structures [38,39]. Moreover, the α-helice and random coil structures content have a tendency to decrease, accompanied by β-sheet and β-turns structures content which have a tendency to increase, while the T2 and T3; T3, T4, and T5 samples were not significantly different (*p* > 0.05). It is possible that the pH was increased with the increase in PBC, a result which caused more buried residues in the protein molecule to be exposed to the water molecules. Then, new hydrogen bonds were formed, leading to the α-helice structure being transformed into β-sheet and β-turns structures during the protein denaturation [19,40]. Thus, this result means that using PBC to replace STPP can change the secondary structure of cooked silver carp batters, and in particular α-helice, β-sheet, and β-turns structures.

## 3. Materials and Methods

### 3.1. Materials

Fresh silver carp (2000 ± 100 g) and pork back fat were purchased from a local farmer’s market (Shangqiu, China). Fresh fish was headed, eviscerated, filleted, minced, and surimi was prepared according to the method of An, You, Xiong, and Yin [41]. Following this, to the surimi was added a sucrose (4%) and sorbitol (4%) mixture as a cryoprotectant, and stored at −20 °C. PBC, STPP, and sodium chloride (analytically pure) were purchased from Tianjin Boddi Chemical Co., Ltd., (Tianjin, China). Spices and sugar were purchased from a local market (Shangqiu, China).

### 3.2. Preparation of the Raw Silver Carp Batters

The recipes of silver carp batters were as follows: silver carp surimi 100 g, pork back fat 20 g, ice water 20 g, white pepper 1 g, garlic powder 1.5 g, sugar 3.5 g, and sodium chloride 1.5 g. Additionally, T1 contained STPP 0.4 g; T2 contained PBC 0.1 g and STPP 0.3 g; T3 contained PBC 0.2 g and STPP 0.2 g; T4 contained PBC 0.3 g and STPP 0.1 g; T5 contained PBC 0.4 g.

Frozen silver carp surimi was thawed to 0–4 °C (core temperature) in a 4 °C freezer. The silver carp batter was produced using a bowl chopper (Stephan UMC-5C, Hamburg, Germany) according to the following method: the thawed silver carp surimi was chopped at 1500 rpm with sodium chloride, PBC or/and STPP, and one-third of the ice water for 30 s; following this, the pork back fat, white pepper, garlic powder, sugar, and one-third of the ice water were added and chopped at 1500 rpm for 30 s; then, this was chopped at 3000 rpm with one-third of the ice water for 60 s. The core temperature of raw silver carp batter was not more than 7 °C. After that, the raw silver carp batter was stored at 4 °C, and used to analyze the pH, emulsion stability, cooking yield, and rheological properties.

### 3.3. Preparation of the Cooked Silver Carp Batters

Approximately 30 g of raw silver carp batter was stuffed in a 50 mL polypropylene tube; then, it was heated at 40 °C in a water bath for 30 min, and heated up to 90 °C for 20 min. Following this, it was cooled immediately in the iced water and stored at 4 °C. The cooked silver carp batter was used to analyze the color, texture profile analysis, and Raman spectroscopy.

### 3.4. pH

Ten grams of sample and 40 mL of distilled water (4 °C) were homogenized at 15,000 rpm for 10 s in an ice bath. The pH of each raw silver carp batter was determined by a digital pH meter.

### 3.5. Emulsion Stability

According to the method of Fernándz-Martín, López-lópez, Cofrades, and Colmenero [42], the emulsion stability was determined, and the total fluid, water, and fat released were obtained. Therein, the total fluid released was expressed as a percentage of the initial sample weight; the water released component (a percentage of the initial sample weight) was determined from the dry matter content of the total fluid released after heating at 105 °C for 16 h. The fat released component (a percentage of the initial sample weight) ignored any minor protein or salt components and was taken as the difference between total fluid released and water released.

### 3.6. Cooking Yield

Cooked silver carp batter was stored at 4 °C for about 12 h; then, the water was cleaned off the surface. The calculation formula was as follows:

Cooking yield = Weight of cooked batter/Weight of raw batter × 100(1)

### 3.7. Color

The center color of the cooked silver carp batter was determined using a colorimeter (CR-400, Minolta Camera Co., Osaka, Japan) with a pulse xenon lamp (the aperture is a diameter of 11 mm), calibrated with a standard white plate (L* = 96.86, a* = −0.15, b* = 1.87). Therein, L*, a*, and b* represented the lightness, redness, and yellowness values. The calculation formula was as follows:

Whiteness = 100 − [(100 − L*)^2^ + a*^2^ + b*^2^]^1/2^ (2)

### 3.8. Texture Profile Analysis

After being stored at 4 °C for about 12 h, the cooked silver carp batter was left at room temperature for 2 h. A cylinder of cooked batter (diameter, 15 mm; height, 20 mm) was cut. Texture profile analysis was conducted by a P/36R probe (Stable Micro System Ltd., Godalming, UK). The pre-test speed was 5.0 mm/s; the test speed was 2.0 mm/s; the post-test speed was 2.0 mm/s; the strain was 50%. The hardness (N), springiness, cohesiveness, and chewiness (N·mm) of the batter were obtained.

### 3.9. Dynamic Rheology Measurement

According to the method of Kang et al. [43], the rheological characteristic of raw silver carp batter was measured by a dynamic rheometer (Haake Mars III, Thermo Scientific, Waltham, MA, USA). Continuous shearing in oscillatory mode was used, and the fixed frequency was 0.1 Hz. Measurements were made at 20 °C for 5 min and then during heating to 90 °C at a rate of 2 °C/min. Changes in storage modulus (G′) were measured.

### 3.10. Raman Spectroscopy

Raman spectroscopy of raw silver carp batter was measured using a modified procedure by Zhu et al. [19]. The spectra were obtained in the range from 400 cm^−1^ to 4000 cm^−1^. The secondary structures of the cooked silver carp batter proteins were determined as percentages of α-helice, β-sheet, β-turn, and random coil or unordered conformations [44,45].

### 3.11. Statistical Analysis

The experiment was repeated four times at different times using different PBC and STPP additive amounts. The data were analyzed through the general linear model (GLM) procedure by SPSS v.20.0. Significant differences (*p* < 0.05) between means were identified by the least significant difference (LSD) procedure.

## 4. Conclusions

This study showed that the use of partial or total PBC to replace STPP affected the color, water- and oil-holding capacity, as well as gel and rheological properties of silver carp batters. The pH, cooking yield, emulsion stability, hardness, springiness, cohesiveness, chewiness, and the G′ at 90 °C of silver carp batters significantly increased with the addition of PBC, which promoted the significant increase in β-sheet and β-turns structures content. With the increase in PBC content, the pH significantly increased and the whiteness significantly decreased. Meanwhile, due to the increase in β-sheet and β-turns structures content and the production of excess carbon dioxide gas, the cooking yield, emulsion stability, texture properties, and the G′ at 90 °C had a tendency to increase and then decrease. Overall, the use of partial PBC to replace STPP can improve the gel characteristics as well as water and oil retention of reduced-phosphate silver carp batter.

## Figures and Tables

**Table 1:** Emulsion stability of the raw silver carp batters made with various amounts of potassium bicarbonate and sodium tripolyphosphate.

Samples	Emulsion Stability (%)		
	Total Fluid Release	Water Released	Fat Released
T1	12.80 ± 0.33 ^a^	9.92 ± 0.28 ^a^	2.63 ± 0.15 ^a^
T2	10.52 ± 0.30 ^b^	8.63 ± 0.30 ^b^	1.87 ± 0.18 ^b^
T3	8.36 ± 0.35 ^c^	6.84 ± 0.25 ^c^	1.39 ± 0.19 ^c^
T4	8.85 ± 0.27 ^c^	6.73 ± 0.27 ^c^	1.51 ± 0.15 ^c^
T5	10.90 ± 0.29 ^b^	8.90 ± 0.25 ^b^	1.92 ± 0.14 ^b^

T1 contained sodium tripolyphosphate 0.4 g; T2 contained potassium bicarbonate 0.1 g and sodium tripolyphosphate 0.3 g; T3 contained potassium bicarbonate 0.2 g and sodium tripolyphosphate 0.2 g; T4 contained potassium bicarbonate 0.3 g and sodium tripolyphosphate 0.1 g; T5 contained potassium bicarbonate 0.4 g. Each value represents the mean ± SD, *n* = 4. ^a–c^ Different parameter superscripts in the table indicate significant differences (*p* < 0.05).

**Table 2:** Texture properties and whiteness of the cooked silver carp batters made with various amounts of potassium bicarbonate and sodium tripolyphosphate.

Sample	Hardness (N)	Springiness	Adhesiveness	Chewiness (N·mm)	Whiteness
T1	47.37 ± 1.27 ^c^	0.853 ± 0.011 ^c^	0.613 ± 0.011 ^c^	24.71 ± 1.27 ^c^	88.71 ± 1.58 ^a^
T2	50.46 ± 1.43 ^b^	0.880 ± 0.015 ^b^	0.639 ± 0.013 ^b^	27.68 ± 1.30 ^b^	84.35 ± 1.66 ^b^
T3	54.13 ± 1.32 ^a^	0.906 ± 0.012 ^a^	0.671 ± 0.010 ^a^	30.65 ± 1.08 ^a^	81.57 ± 1.46 ^c^
T4	53.91 ± 1.13 ^a^	0.911 ± 0.012 ^a^	0.670 ± 0.009 ^a^	31.10 ± 1.21 ^a^	77.82 ± 1.93 ^d^
T5	51.22 ± 1.36 ^b^	0.877 ± 0.014 ^b^	0.627 ± 0.014 ^b^	26.83 ± 1.17 ^b^	74.26 ± 1.63 ^e^

T1 contained sodium tripolyphosphate 0.4 g; T2 contained potassium bicarbonate 0.1 g and sodium tripolyphosphate 0.3 g; T3 contained potassium bicarbonate 0.2 g and sodium tripolyphosphate 0.2 g; T4 contained potassium bicarbonate 0.3 g and sodium tripolyphosphate 0.1 g; T5 contained potassium bicarbonate 0.4 g. Each value represents the mean ± SD, *n* = 4. ^a–e^ Different parameter superscripts in the table indicate significant differences (*p* < 0.05).

**Table 3:** Percentages of protein secondary structures (α-helice, β-sheet, β-turns, and random coil) of the cooked silver carp batters made with various amounts of sodium tripolyphosphate and potassium bicarbonate.

Sample	α-Helice	β-Sheet	β-Turn	Random Coil
T1	37.81 ± 1.63 ^a^	27.65 ± 1.20 ^c^	22.36 ± 0.61 ^c^	13.05 ± 0.27 ^a^
T2	34.07 ± 1.70 ^b^	30.72 ± 1.44 ^b^	24.20 ± 0.53 ^b^	11.82 ± 0.25 ^b^
T3	32.85 ± 1.86 ^bc^	32.02 ± 1.36 ^ab^	25.37 ± 0.41 ^ab^	10.66 ± 0.31 ^b^
T4	31.18 ± 2.03 ^c^	33.46 ± 1.51 ^a^	26.63 ± 0.44 ^a^	10.11 ± 0.36 ^b^
T5	30.33 ± 1.96 ^c^	33.70 ± 1.33 ^a^	27.48 ± 0.50 ^a^	9.67 ± 0.29 ^bc^

T1 contained sodium tripolyphosphate 0.4 g; T2 contained potassium bicarbonate 0.1 g and sodium tripolyphosphate 0.3 g; T3 contained potassium bicarbonate 0.2 g and sodium tripolyphosphate 0.2 g; T4 contained potassium bicarbonate 0.3 g and sodium tripolyphosphate 0.1 g; T5 contained potassium bicarbonate 0.4 g. Each value represents the mean ± SD, *n* = 4. ^a–c^ Different parameter superscripts in the figure indicate significant differences (*p* < 0.05).

## Data Availability

Research data are not shared.
